# Exploring the Connection between Sleep Disorders, Emotional Distress, and Quality of Life in Functional Dyspepsia

**DOI:** 10.62641/aep.v53i2.1947

**Published:** 2025-03-05

**Authors:** Jijun Xiong, Zheng Wang, Gang Chen, Jianjun Fu

**Affiliations:** ^1^Department of Gastroenterology, Gong An County People’s Hospital, 434300 Jingzhou, Hubei, China

**Keywords:** sleep disorders, functional dyspepsia, emotional distress, quality of life

## Abstract

**Objective:**

This study aimed to explore the factors influencing sleep disorders in patients with functional dyspepsia.

**Methods:**

A total of 100 patients with functional dyspepsia admitted to Gong An County People’s Hospital from 2020 to 2021 were selected. According to the Pittsburgh Sleep Quality Index (PSQI), those with a score ≥8 were classified as the occurrence group, whereas those with a score <8 were classified as the non-occurrence group. Clinical and disease characteristics of patients were collected. Logistic regression analysis was used to identify influencing factors. The emotional distress and quality of life of patients with different severities of sleep disorders were compared. Pearson’s correlation was used to identify the relationship between the degree of sleep disorders and various indicators.

**Results:**

Out of 100 patients with functional dyspepsia, 58 (58.00%) had varying degrees of sleep disturbance. Logistic regression analysis showed that factors measured by the Self-Rating Anxiety Scale (SAS) (odds ratio [OR] = 3.088, *p* = 0.007), Self-Rating Depression Scale (SDS) (OR = 3.268, *p* = 0.005), Perceived Stress Scale (PSS) (OR = 2.659, *p* = 0.019), and Functional Digestive Disorders Quality of Life (FDDQL) questionnaire (OR = 2.591, *p* = 0.022) were the main factors influencing sleep disturbance. Pearson correlation analysis suggested that SAS (r = 0.677, *p* < 0.001), SDS (r = 0.623, *p* < 0.001), and PSS (r = 0.550, *p* < 0.001) scores were positively correlated with the severity of sleep disturbance, whereas FDDQL (r = –0.623, *p* < 0.001) score was negatively correlated with the severity of sleep disturbance.

**Conclusion:**

Functional dyspepsia patients are prone to varying degrees of sleep disorders, which are closely related to emotional distress and quality of life. Clinical interventions can be developed in advance to stabilize patient emotions and improve their quality of life.

## Introduction

Functional dyspepsia is a common functional gastrointestinal disorder, with an estimated prevalence of 20%–25% [1]. Patients typically present with symptoms such as early satiety, belching, and abdominal pain, often characterized by recurrent episodes with a difficult-to-treat nature. The etiology of functional dyspepsia is closely associated with factors such as *Helicobacter pylori* infection, dietary habits, and lifestyle choices. Sleep disturbances are frequently observed in functional dyspepsia patients, and alterations in sleep quality may adversely affect their physical and mental well-being. The prevalence of sleep disorders among individuals with functional dyspepsia is notably high, with insomnia rates reaching up to 61% [2]. Sleep disorders not only impact the quality of sleep but may also led to symptoms such as fatigue and compromised immunity, exacerbating the severity of the condition and negatively impacting patients’ quality of life [3].

Research has revealed a close correlation between sleep disorders and the pathogenesis of functional dyspepsia. This association may be attributed to abnormalities in neural regulation, where aberrant neural signals directly stimulate the central nervous system, thereby increasing the risk of sleep disturbances [4]. Furthermore, adverse sleep environments have been found to exert a negative impact on the sleep quality of individuals with functional dyspepsia. Common environmental factors such as noise, light, and temperature disturbances could disrupt sleep, exacerbating the severity of sleep disorders [5].

In addition to neural and environmental factors, the relationship between functional dyspepsia and emotional distress is also of significant interest. A study found that some patients may experience feelings of depression and anxiety due to the recurrence of symptoms and persistent stress, directly affecting their sleep quality [6]. Prolonged feelings of anxiety and depression may lead to difficulties in falling asleep and increased nocturnal awakenings, further compromising the overall quality of sleep. Moreover, emotional distress may result in negative emotional experiences during sleep, such as fear, unease, or depression, further impacting the stability and continuity of sleep quality. This intricate interplay makes individuals with functional dyspepsia more susceptible to falling into a vicious cycle when faced with sleep disturbances.

If we can fully understand the factors influencing sleep disorders in patients with functional dyspepsia, we can therefore provide in-depth theoretical and empirical support for the development of more accurate rehabilitation programs and for improving patients’ overall health status. Accordingly, the aim of this study is to provide a comprehensive assessment of the factors influencing sleep disorders in patients with functional dyspepsia through a cross-sectional survey, as well as to explore the relationship between sleep disorders and emotional distress and quality of life. Specific guidance and strategies can be provided for future rehabilitation management and clinical practice.

## Materials and Methods

### General Information

A total of 100 patients with functional dyspepsia admitted to Gong An County People’s Hospital from 2020 to 2021 were selected as the observation group. This study was approved by the ethics committee of Gong An County People’s Hospital. All procedures were conducted in accordance with the Declaration of Helsinki.

The inclusion criteria were as follows: (1) have a diagnosis of functional dyspepsia consistent with Rome IV-Functional GI Disorders and the 10th revision of the International Classification of Diseases; (2) have basic listening, speaking, reading, and writing skills, and be fully coherent; and (3) have complete patient clinical data.

The exclusion criteria were as follows: (1) digestive system symptoms caused by liver disease or kidney disease; (2) digestive system symptoms caused by drugs or other substances; (3) digestive system symptoms caused by excessive alcohol or drug abuse; (4) digestive system symptoms such as abdominal pain, diarrhea, nausea, and vomiting caused by mental disorders or psychological problems; (5) food intolerance or allergy symptoms; and (6) symptoms caused by infection of the digestive system or parasitic infection.

### Methods

(1) We collected the patients’ general social and disease-related characteristics, including sex, age, marital status, body mass index, and severity of illness (Acute Physiology and Chronic Health Evaluation [APACHE] Ⅱ, mild: <30; moderate-to-severe: 30–71 points), course of disease, monthly income, education level, employment status, smoking history, drinking history, history of mental illness, family history of mental illness, and complications.

(2) The sleep conditions of the patients in the past month were evaluated using the Pittsburgh Sleep Quality Index (PSQI) [7]. A total of 18 items comprising seven components were included. Each component was scored according to the 3-point Likert Scale scoring method. The total score was 0–21 points, with <8 points indicating no sleep disorder, 8–13 points indicating a mild disorder, 14–18 points indicating a moderate disorder, and 19–21 points indicating a severe disorder. The lower score the better the sleep quality. The overall Cronbach’s α coefficient of the scale was 0.936, indicating good reliability and validity.

(3) Emotional distress was evaluated using the Self-Rating Anxiety Scale (SAS), the Self-Rating Depression Scale (SDS) [8], and the Perceived Stress Scale (PSS) [9]. ① The SAS and SDS included 20 items. They primarily evaluated patients’ subjective feelings of anxiety and depression. The total score was 80 points, with an SAS score >50 points indicating the presence of anxiety. An SDS score >53 points indicated depression, and a higher score indicated more serious depression. The overall Cronbach’s α coefficients of the scale were 0.879 and 0.912, respectively, showing good reliability and validity. ② The PSS primarily evaluated the patient’s individual stress level using 14 items according to the 5-point Likert Scale scoring method. The full score was 14–70 points, and a higher score corresponded with more obvious psychological stress. The overall Cronbach’s α coefficient of the scale was 0.891, showing good reliability and validity.

(4) The patients’ quality of life was assessed using the Functional Dyspepsia Quality of Life questionnaire (FDDQL) [10], which includes eight aspects: diet, health, sleep, feelings of worry, discomfort, stress, and treatments. The FDDQL had 43 items and a total of 100 points. A higher score corresponded with a better quality of life. The overall Cronbach’s α coefficient of the scale was 0.922, showing good reliability and validity.

### Observation Indicators

The sleep status of all patients was observed, and the clinical data of each group were compared. The factors influencing the occurrence of sleep disorders in patients with functional dyspepsia were analyzed and compared with the emotional distress and quality of life of patients having different severity levels of sleep disorders. The relationship between the degree of sleep disorders and SAS, SDS, PSS, and FDDQL scores was also explored.

### Statistical Analyses

IBM SPSS Statistics for Windows version 25.0 (IBM Corp., Armonk, NY, USA) was used for data analyses. Count data were expressed as the number of cases (n) by the χ^2^ test. Measurement data conforming to normal distribution were expressed as ($\bar{x}$ ± s), and the Student’s *t*-test was used. The F-test was used between multiple groups. Logistic regression equations were selected for the items that differed between the two groups, and the factors affecting sleep disorders in patients with functional dyspepsia were analyzed by using the presence or absence of sleep disorders as the dependent variable and clinical data as the independent variable. Variables with *p* < 0.05 were gradually screened as the standard. Spearman’s correlation analyses were performed to determine the relationship between the severity of sleep disorders and SAS, SDS, PSS, and FDDQL scores. *p* < 0.05 indicated that the difference was statistically significant.

## Results

### PSQI Scores of All Patients

A total of 100 patients with functional dyspepsia were included in this study. The total PSQI score was 11.92 ± 3.73. Forty-two cases (42.00%) had no sleep disorders and comprised the non-occurrence group; 26 were mild cases (26.00%), 23 were moderate (23.00%), and nine were severe (9.00%), totaling 58 patients in the occurrence group. Details are shown in Table 1.

**Table 1.** **PSQI scores for each entry and total score for all patients**.

PSQI score		($\bar{x}$ ± s)/n (%)
Sleep latency		1.85 ± 0.51
Sleep disorders		2.03 ± 0.47
Subjective sleep quality		1.62 ± 0.44
Daytime dysfunction		1.55 ± 0.31
Persistence of sleep		1.70 ± 0.55
Use of sleep medications		1.48 ± 0.39
Habitual sleep efficiency		1.69 ± 0.52
Total score		11.92 ± 3.73
	<8 points	42 (42.00%)
	8–13 points	26 (26.00%)
	14–18 points	23 (23.00%)
	19–21 points	9 (9.00%)

Note: PSQI, Pittsburgh Sleep Quality Index.

### Comparison of General Data of Each Group

Significant differences existed in body mass index, monthly income, education level, SAS, SDS, PSS, and FDDQL scores between the occurrence and non-occurrence groups (*p* < 0.05). Details are shown in Table 2.

**Table 2.** **Comparison of general data of each group (n = 100)**.

Clinical data		n	Occurrence group (n = 58)	Non-occurrence group (n = 42)	χ ^2^	*p*
Gender	Male	44	27	17	0.365	0.546
	Female	56	31	25		
Age (years)	≤60	64	36	28	0.223	0.636
	>60	36	22	14		
Marital status	Married	62	39	23	1.610	0.204
	Unmarried	38	19	19		
Body mass index (kg/m^2^)	≤25	47	21	26	6.458	0.011
	>25	53	37	16		
Severity of illness	Mild	54	30	24	0.288	0.592
	Moderate-to-severe	46	28	18		
Duration of disease (years)	≤3	39	19	20	2.261	0.133
	>3	61	39	22		
Monthly income (CNY)	≤5000	55	38	17	6.172	0.013
	>5000	45	20	25		
Degree of education	Junior high school and below	49	35	14	7.112	0.008
	High school and above	51	23	28		
Situation of employment	On the job	77	41	36	3.105	0.078
	Unemployment	23	17	6		
History of smoking	Yes	59	32	27	0.836	0.360
	No	41	26	15		
History of alcohol consumption	Yes	50	28	22	0.164	0.685
	No	50	30	20		
A history of mental illness	Yes	29	16	13	0.134	0.714
	No	71	42	29		
Family history of mental illness	Yes	21	11	10	0.345	0.557
	No	79	47	32		
Comorbidity	Yes	63	35	28	0.418	0.518
	No	37	23	14		
SAS score	≤50	46	20	26	7.374	0.007
	>50	54	38	16		
SDS score	≤53	43	18	25	8.067	0.005
	>53	57	40	17		
PSS score	≤40	48	22	26	5.609	0.018
	>40	52	36	16		
FDDQL score	≤60	55	37	17	5.332	0.021
	>60	45	21	25		

Note: SAS, Self-Rating Anxiety Scale; SDS, Self-Rating Depression Scale; PSS, Perceived Stress Scale; FDDQL, Functional Digestive Disorders Quality of Life; The exchange rate is 1 USD = 6.48 CNY.

### Multivariate Analysis of Factors Influencing Sleep Disorders in Patients with Functional Dyspepsia

Logistic regression analysis showed that SAS, SDS, PSS, and FDDQL scores were influencing factors of sleep disorders in patients with functional dyspepsia. All odds ratio (OR) values were >1. Details are shown in Table 3.

**Table 3.** **Multivariate analysis of factors affecting sleep disorders in patients with functional dyspepsia**.

Influencing factors	Assignment of value	Regression coefficient	SE	Wald value	*p*	OR value	95% CI
SAS score	0=SAS ≤50,	1.127	0.421	7.169	0.007	3.088	1.353–7.047
	1=SAS >50						
SDS score	0=SDS ≤53,	1.184	0.424	7.817	0.005	3.268	1.425–7.495
	1=SDS >53						
PSS score	0=PSS ≤40,	0.978	0.417	5.491	0.019	2.659	1.173–6.026
	1=PSS >40						
FDDQL score	0=FDDQL >60,	0.952	0.416	5.225	0.022	2.591	1.145–5.861
	1=FDDQL ≤60						

Note: SAS, Self-Rating Anxiety Scale; SDS, Self-Rating Depression Scale; PSS, Perceived Stress Scale; FDDQL, Functional Digestive Disorders Quality of Life; SE, standard error; OR, odds ratio; CI, confidence intervals.

### Emotional Distress and Quality of Life at Different Levels of Sleep Disorders

Significant differences existed in SAS, SDS, PSS, and FDDQL scores among patients with different severity levels of sleep disorders (*p* < 0.05). Details are shown in Table 4.

**Table 4.** **Emotional distress and quality of life at different levels of sleep disorders (score)**.

Sleep disorder	SAS	SDS	PSS	FDDQL
Mild disorder (n = 26)	46.35 ± 5.11	50.46 ± 5.32	37.23 ± 4.10	73.12 ± 8.16
Moderate impairment (n = 23)	52.87 ± 5.59	56.17 ± 5.80	40.96 ± 4.53	67.26 ± 7.77
Serious disorder (n = 9)	60.11 ± 6.13	63.00 ± 6.46	45.22 ± 4.99	54.22 ± 6.91
F	23.030	17.550	11.960	19.570
*p*	<0.001	<0.001	<0.001	<0.001

Note: SAS, Self-Rating Anxiety Scale; SDS, Self-Rating Depression Scale; PSS, Perceived Stress Scale; FDDQL, Functional Digestive Disorders Quality of Life.

### Correlation between the Degree of Sleep Disturbance and Emotional Distress and Quality of Life

Pearson’s correlation showed a positive correlation with SAS, SDS, and PSS score, as well as a negative correlation with FDDQL score (*p* < 0.05). Details are shown in Table 5.

**Table 5.** **Correlation between the degree of sleep disturbance and emotional distress and quality of life**.

Norm	*r*	*p*	95% CI
SAS	0.677	<0.001	0.5069, 0.7958
SDS	0.623	<0.001	0.4348, 0.7592
PSS	0.550	<0.001	0.3399, 0.7077
FDDQL	–0.623	<0.001	–0.7590, –0.4344

Note: Setting Mild Impairment: 1; Moderate Impairment: 2; Severe Impairment: 3; SAS, Self-Rating Anxiety Scale; SDS, Self-Rating Depression Scale; PSS, Perceived Stress Scale; FDDQL, Functional Digestive Disorders Quality of Life; CI, confidence intervals.

## Discussion

Functional dyspepsia is a type of common functional gastrointestinal disease. Patients usually suffer from upper abdominal pain, as well as digestive tract symptoms such as vomiting and early fullness. Other symptoms include anxiety, depression, and even sleep disorders. This finding is primarily due to the close relationship between the disease mechanism and the living environment and mental stress, in addition to dietary problems and other problems related to the disease [11]. Clinical findings reveal that functional dyspepsia patients often experience sleep disorders primarily because the disease can lead to parasomnias and other problems. Many factors affect sleep disorders in patients with functional dyspepsia, so early understanding and timely intervention are the keys to treatment. Andreev *et al*. [12] found that the prevalence of sleep disorders in patients with functional dyspepsia is 23.95%. Yamamoto *et al*. [13] also demonstrated a positive correlation between sleep disorders and the prevalence of functional dyspepsia. Our results showed that patients with functional dyspepsia have varying degrees of sleep disorders, with over half of them experiencing mild, moderate, or severe sleep problems. Patients with functional dyspepsia are also often disturbed by gastrointestinal motility. This phenomenon causes the central nervous system to be in an excited state. Brain-gut peptides, which are common peptides in the gastrointestinal tract and nervous system, can regulate gastrointestinal sensation and movement to control appetite. They play important roles in the central nervous system control of gastrointestinal function, and also greatly impact the occurrence and development of functional dyspepsia. Sleep disorders are also related to brain-gut peptides such as melatonin, somatotropin and leptin, which can lead to functional dyspepsia and increase the risk of sleep disorders [14]. Severe epigastric pain and burning may further cause disturbances in initiating and maintaining sleep, which in turn may aggravate functional dyspepsia. Therefore, patients with functional dyspepsia are prone to sleep disorders and even suffer from parasomnia. If not promptly improved by intervention measures, the mental state of patients may be directly affected and further aggravate their functional dyspepsia. This phenomenon forms a vicious circle and has thus elicited the attention of clinical researchers.

The present study revealed that anxiety was one of the factors influencing the occurrence of sleep disorders in patients with functional dyspepsia. More severe sleep disorders corresponded with a deeper level of anxiety. Caballero-Mateos [15] stated that anxiety is a common psychological symptom in patients with inflammatory bowel disease, especially patients with functional dyspepsia, with the prevalence of anxiety symptoms being approximately 38.5%. A higher degree of anxiety corresponds to a higher incidence of sleep disorders primarily because anxiety is an unpleasant subjective emotional experience. At the mental level, the abnormal hormone secretion and excessive activation of sympathetic nervous system caused by anxiety are mechanisms of sleep disorders. Anxiety is therefore an important factor influencing sleep disorders. Koreshkina [16] found consistent results, in that anxiety disorders are a risk factor for sleep disorders.

Soboka *et al*. [17] reported that the prevalence of depression in dyspepsia patients is high, reaching approximately 22.6%. A more serious risk of sleep disorders means greater depression, primarily because patients with depression may have directly affected brain norepinephrine, serotonin, and neurotransmitter levels, and elevated cortisol concentrations at night. Their sleep rhythm changes, so as to increase the number of awakenings, reducing total sleep time and further increasing the risk of sleep disturbances. The present study also found that depression was a risk factor for the development of sleep disorders in patients with functional dyspepsia. The degree of depression deepened with increased degree of sleep disorder. This finding is in line with the conclusion of Lu *et al*. [18], who found that the occurrence and severity of sleep disorders are significantly correlated with depression.

Using the stress scale, Tshabalala *et al*. [19] found that 67.5% of dyspepsia patients suffer from stress. More severe stress corresponds with a greater likelihood of developing sleep disorders. PSS is an influential factor in the occurrence of sleep disorders in patients with functional dyspepsia, especially in those with severe sleep disorders who have greater psychological stress. Dresp-Langley B [20] pointed out that stress is the main risk factor for sleep disorders, and can directly affect the regulation function of the hypothalamic–pituitary–adrenal axis of the individual, leading to sleep disorders.

Goyal *et al*. [21] pointed out that quality of life is significantly decreased in patients with functional dyspepsia and severe indigestion, compared with patients with mild indigestion. A poorer quality of life means a higher risk of sleep disorders. This finding is similar to the results of the present study, in which quality of life and severity of sleep disorders were shown to be negatively correlated. Xiao *et al*. [22] reported that sleep disorders have become some of the most important factors affecting people’s lives. The reason is that quality of life is usually affected by external and internal factors. External factors include social recognition, socioeconomic status, and quality of medical care, whereas internal factors refer to the type of disease, severity of disease, comorbidities, anxiety, and depression [23]. Patients with functional dyspepsia may experience abdominal pain and bloating at night, which can easily lead to difficulties in falling asleep. These patients need to consistently follow dietary restrictions, such as avoiding spicy foods and alcohol, which can negatively affect their quality of life and make them resistant to change, thereby further lowering their quality of sleep. Coupled with the recurrence of the disease, these conditions may increase the family’s financial burden, which can lead to increased pressure on work and life in general, which can lead to the patients’ poor quality of sleep. However, emotional regulation difficulties such as anxiety, depression, and stress can greatly affect the quality of life and have become an important factor in the occurrence of sleep disorders [24, 25].

## Conclusion

Patients with functional dyspepsia are prone to sleep disorders. Many factors affect the occurrence of sleep disorders, and are closely related to emotional distress and quality of life. This phenomenon warrants clinical research attention so that early intervention measures can be taken. The goal is to stabilize patients’ emotions and improve their quality of life.

## Availability of Data and Materials

The original contributions presented in the study are included in the article. Further inquiries can be directed to the corresponding author.
